# A review of the resistome within the digestive tract of livestock

**DOI:** 10.1186/s40104-021-00643-6

**Published:** 2021-11-11

**Authors:** Tao Ma, Tim A. McAllister, Le Luo Guan

**Affiliations:** 1 Key laboratory of Feed Biotechnology of the Ministry of Agriculture, Institute of Feed Research, Chinese Academy of Agricultural Sciences, Beijing, 100081 China; 2 Department of Agricultural, Food and Nutritional Science, University of Alberta, T6G2P5, Edmonton, AB Canada; 3 Lethbridge Research and Development Centre, Lethbridge, AB T1J 4P4 Canada

**Keywords:** Antimicrobial resistance, Antimicrobial resistance gene, Digestive tract, Food-producing animal, Metagenomic sequencing, Resistome

## Abstract

Antimicrobials have been widely used to prevent and treat infectious diseases and promote growth in food-production animals. However, the occurrence of antimicrobial resistance poses a huge threat to public and animal health, especially in less developed countries where food-producing animals often intermingle with humans. To limit the spread of antimicrobial resistance from food-production animals to humans and the environment, it is essential to have a comprehensive knowledge of the role of the resistome in antimicrobial resistance (AMR), The resistome refers to the collection of all antimicrobial resistance genes associated with microbiota in a given environment. The dense microbiota in the digestive tract is known to harbour one of the most diverse resistomes in nature. Studies of the resistome in the digestive tract of humans and animals are increasing exponentially as a result of advancements in next-generation sequencing and the expansion of bioinformatic resources/tools to identify and describe the resistome. In this review, we outline the various tools/bioinformatic pipelines currently available to characterize and understand the nature of the intestinal resistome of swine, poultry, and ruminants. We then propose future research directions including analysis of resistome using long-read sequencing, investigation in the role of mobile genetic elements in the expression, function and transmission of AMR. This review outlines the current knowledge and approaches to studying the resistome in food-producing animals and sheds light on future strategies to reduce antimicrobial usage and control the spread of AMR both within and from livestock production systems.

## Introduction

It is estimated that the world population will reach 9.1 billion in 2050, among which 70% will be urbanites [1]. Concomitantly, the consumption of meat and eggs, as well as dairy products, is predicted to increase by 73% and 58%, worldwide respectively, by 2050 [2]. In order to meet the increasing demand for animal food products, strategies should be implemented to improve growth and efficiency in ruminants, swine, and chickens. In fact, meat production in low- and middle-income countries such as Africa, Asia, and South America have increased by 68%, 64%, and 40% respectively, since 2000 [3], which is largely due to the adoption of of intensive production systems.

In such systems, antimicrobials play a vital role in increasing the health and production efficiency of livestock [4]. Since the 1950s, antimicrobials have been used in livestock and poultry production to prevent and treat diseases and to improve the feed conversion efficiency and promote growth [5]. The global total use of antimicrobials for cattle, pigs and chickens will increase from approximately 63,000 tons in 2010 to approximately 105,000 tons by 2030, an increase of up to 67% [6]. In European Union, 8,927 tons of antimicrobials were used in food-producing animals in 2004 [7]. In the US, sub-therapeutic doses of antimicrobials used in food-producing animals reached approximately 14,600 tons in 2012 [8]. In China, the world’s largest producer and consumer of antimicrobials, 29,774.09 tons of antimicrobials were used in animal husbandry in 2018, with more than half of this amount used to promote animal growth [9, 10]. Despite their benefits, there is growing evidence that large-scale use of antimicrobials in food-producing animals selects for antimicrobial resistance (AMR) bacteria in livestock [11–13]. The selection for AMR bacteria not only increases morbidity and mortality in food-producing animals but also increases the risk of transmission of AMR bacteria to human beings [14–16]. This is because the antimicrobial resistance genes (ARGs) of the bacteria in the digestive tract of food-producing animals can be transferred to bacteria that can come in contact with humans either directly or from the environment. In this regard, the World Health Organization called on its member countries to reduce the use of veterinary antimicrobial drugs in 2017 [17, 18]. Therefore, AMR is one of the most urgent challenges facing the world currently, posing a threat to health care and food safety.

Efforts have been made to limit the potential spread of AMR from food-producing animals to human beings and environment. For example, many European countries have banned the use of antimicrobials in farm animals for ‘non-therapeutic’ purposes [19]. Recently, the Chinese government launched a regulation to withdraw medicated feed additives in accordance with the National Action Plan to Combat Antimicrobial Resistance from Animal Resources (2017–2020) [20–22]. In addition to these policies, several strategies have been developed (e.g. the use of bacteriophages, antimicrobial peptides, or vaccines) which may be promising to replace the use of antimicrobials in food-producing animals, which have been extensively reviewed [23, 24] While these strategies are crucial to restrict the prevalence of AMR in food-producing animals, ARGs can still be detected in the animal production systems even if no antimicrobials are administered [25]. In order to further reduce the spread of ARGs from food-producing animals to humans or the environment, it is essential to clarify which ARGs are carried by the microbes (bacteria or archaea) inhabiting the digestive tract of food-producing animals. In recent years, with the development of next-generation sequencing (NGS) techniques, studies have enabled the characterization of a collection of ARGs, termed the resistome [26, 27], in a variety of environments including water, soil, as well as the digestive tract of humans and livestock [28–30]. This approach has greatly expanded the scope of ARG monitoring compared with traditional culture and/or polymerase chain reaction (PCR)-based techniques [31, 32]. In this review, we first introduce methods, pipelines (e.g. read- and assembly-based approaches), resources/tools, and databases for resistome identification using shotgun metagenomic sequencing techniques. We then summarize the findings on the profiles and abundance of resistome, as well as define the factors (e.g. dietary composition or use of antimicrobials) that influence the resistome in the digestive tract of swine, poultry, and ruminants (cattle and sheep). Finally, we propose future research advancements in the application of long-read sequencing in resistome analysis, role of mobile genetic elements in resistome development, and expression profiles of the resistome**.** We also propose host, microbial, and environmental factors that may explain the effect of antimicrobial use on resistome profiles in the digestive tract of food-producing animals, with an aim to develop strategies to control the spread of ARGs from food-producing animals to humans.

### Metagenomic-based approach for resistome characterization

Traditionally, two state-of-the-art approaches have been used to detect ARGs within their bacterial host. One approach is based on culturing, antimicrobial susceptibility testing, and polymerase chain reaction. However, this approach only applies to culturable bacteria and does not enable the discovery of distantly related or unknown elements [33]. The other approach is based on whole genome sequencing, which enables the detection of complete genome in bacteria including the ARGs that it may carry [33, 34]. However, this approach can only identify ARGs of in collected isolates and may not reflect the complexity of the resistome *in vivo*, as the digestive tract of humans and animals harbors a complex and dynamic bacterial population. The advent and development of NGS technologies has enabled and accelerated research of environmental microbiome using shotgun metagenomics [35, 36], which also allows the analysis of the whole genome as well as identification of unknown genetic elements of both culturable and unculturable microbial species [37, 38]. More recently, shotgun metagenomics has expanded our abilities to comprehensively investigate ARGs, as a result of an increase in the availability of bacterial genome databases as well as decrease in sequencing costs.

#### Estimation of sample size

Prior to initiating an experiment, it is crucial for a researcher to estimate the statistical power to determine the sample size needed for a resistome studies. This is important and needed to test the research hypothesis adequately and draw meaningful conclusions [39]. To conduct power calculations in microbiome research, methods such as t-test, analysis of variance, χ^2^ test, and the Dirichlet-Multinomial model can be applied [40]. Some web-based pipelines are also available for sample size and power calculation in microbiome studies [41, 42]. However, the sample sizes have not been evaluated in most resistome studies in food-producing animals. One reason could be that it may be difficult to perform a formal sample size evaluation for specific food-producing animals, as the related studies are highly limited and the variation among individuals for a population is largely unknown.

#### Sample preparation

Another key to the success of metagenomic analysis is to obtain high-quality DNA for unbiased interpretation of microbiota in the digestive tract of food-producing animals. Therefore, proper sampling and storage processes need to be taken into account during the design of resistome studies. Under practical conditions, it is not feasible to collect and process samples on the same day, as samples are usually collected at different time points and stored at −80°C for future analysis. In this regard, appropriate methods for sample storage and DNA extraction are essential to accurately represent the gut microbiota using NGS technologies [43]. A recent study showed that sample storage reagents and DNA extraction methods interactively impact the recovery of gut microbiota from chickens and pigs [44]. Consequently, standardization of protocols for sample storage and DNA extraction are needed in the study of gut microbiota in food-producing animals.

#### Quality control of metagenomic reads

The most crucial step before the analysis of resistome is the quality control (QC) of metagenomic reads. Workflows to conduct QC of metagenomic analysis have been extensively reviewed by Li et al. [36], which mainly includes trimming low-quality bases and residual artificial sequences as well as depleting host DNA. Trimmomatic [45] and Cutadapt [46] are frequently used tools for adapter trimming and read filtering. Non-microbial host DNA can be a major concern for sequencing-based microbiome analysis, especially for samples that are low in microbial biomass [47, 48]. It has been shown that samples with high amounts of host DNA coupled with reduced sequencing depths lower the sensitivity of microbiome profiling within metagenomic datasets [49]. Host DNA can be depleted either experimentally or bioinformatically. For example, the use of MolYsis complete5 kit was shown to effectively remove host DNA in bovine milk samples [48]. In addition to experimental methods, several bioinformatic tools/resources such as MG-RAST [50] and TopHat2 [51] can be further used to filter bovine DNA and RNA sequences from metagenomic or metatranscriptomic datasets [52, 53]. While MG-RAST is a web-based pipeline, TopHat2 can be ran on a desk top computer, with both approaches using Bowtie aligner [54] to map contaminated reads against a host genome database. Other pipelines such as MetaWRAP [55] and Sunbeam [56] that incorporate Trimmomatic or Bowtie aligner can also be used for QC and removal of host-derived reads.

#### Assembly-based analysis vs. Read-based analysis

**Fig. 1:**
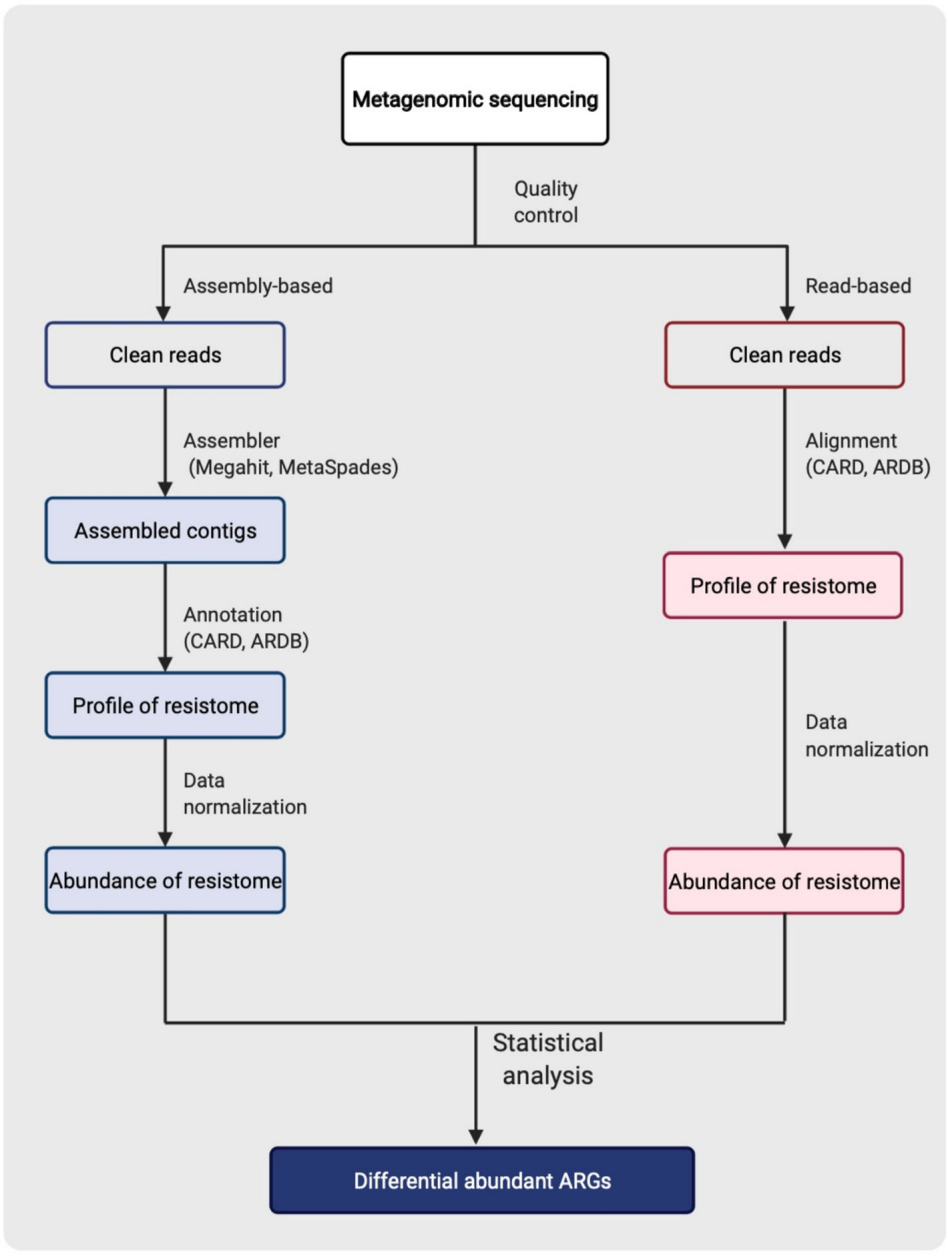
Pipelines for the analysis of resistome based on metagenomic sequencing (Created with BioRender.com)

To analyze the resistome based on shotgun metagenomics, the short sequence reads generated by any sequencing platforms such as Illumina can either be directly mapped to reference databases (defined as reads-based methods), or first assembled into contigs and then annotated though comparison with reference databases (defined as assembly-based methods) [57]. For both approaches, high-quality data and reliable bioinformatics pipelines are needed. Here we provide an overview of the major steps of metagenomic-based resistome analysis (Fig. 1).

For assembly-based methods, the first step is to assemble the post-QC reads into contigs. This can be performed using De Bruijn graph-based assemblers, with MEGAHIT [58] and MetaSPAdes [59] being most frequently used [60]. A recent review described the protocols of these assemblers in detail [61]. It should be noted that multiple biological (e.g. sample origin, biomass, and representation) and technical (e.g. sequence quality, depth, and platform) factors may affect the performance of an assembler’s ability to generate high-quality contigs [62]. Thus, different assemblers should be tested on a subset of samples to identify the optimal procedure for a given dataset. For read-based approaches, ARGs can be detected either by aligning reads directly to the reference databases using aligners such as Bowtie2 [54] or Burrows-Wheeler aligner (BWA) [63], or by splitting reads into k-mers before mapping them to reference databases [57]. Specific tools such as Short Read Sequencing Typing (SRST2) [64] can be used to identify AMR in samples with insufficient reads to perform *de novo* assembly. More recently, tools such as Antimicrobial Resistance Identification By Assembly (ARIBA) [65] and graphing Resistance Out Of meTagenomes (GROOT) [66], have been developed that can efficiently map large-scale sequence datasets.

It should be noted that there is no consensus on which approach (read- vs. assembly-based) is more accurate and both have advantages and disadvantages [67]. For example, read-based approaches are generally faster and less computationally demanding as compared with assembly-based methods, as they bypass *de novo* assembly, protein-coding gene prediction and pairwise alignment to public databases. However, read based approaches lack the positional information required to analyze upstream and downstream factors of identified resistance genes. In contrast, assembly-based methods can construct whole genomes or large contigs with protein-coding genes and regulatory sequence information, enabling generation of a surrounding genomic context. This enables the analyses of co-associated genes and biological pathways that could play a role in the regulation of ARGs. Nevertheless, assembly-based methods are computationally demanding, time consuming and requires deeper genomic coverage to avoid information loss [68]. In this regard, the profiles of resistome among studies need to be interpreted and compared with caution if different analysis approach are used.

#### ARG reference database

In addition to data processing and analysis approaches, the prediction accuracy of ARGs also relies on the integrity of the reference databases. The ARG reference databases can be divided into two types based on their features. For example, databases such as Antibiotic Resistance Genes Database (ARDB, not curated since 2009) [69], ResFinder [70], and Comprehensive Antibiotic Resistance Database (CARD, also contains all data from ARDB) [71] have been used to detect all known sequenced ARGs, while those such as Antibiotic Resistance Genes Online (ARGO) are specifically used to detect only β-lactam and vancomycin resistance genes [72]. Secondly, the criteria for entry in the reference databases are different: while CARD requires the entries must have been published in the scientific literature [71], ResFinder lacks this requirement [70]. Thirdly, the types of entries differ between reference databases. Most reference databases include all drug resistance genes, and only a few reference databases also include drug resistance genes that arise as a result of chromosomal mutations (e.g. MUBII-TB-DB [73] and PointFinder [74]). Finally, entry format also differs among reference databases (e.g. fasta or json, etc.), download permissions, and regular maintenance frequency [75]. Therefore, it is necessary to have a full understanding of these distinguishing characteristics when selecting a suitable database for resistome analysis.

#### Annotation of ARGs and data process

For assembly-based methods, the metagenomic-assembled contigs are annotated for resistance determinants by predicting protein-coding regions on contigs, which are then compared against AMR reference databases using similarity-based search tools such as Basic local alignment search tool (BLAST) [76] or DIAMOND [77]. For read-based analysis, post-QC reads are directly aligned to AMR reference databases using alignment tools as mentioned above for the characterization of ARGs. The annotated ARG can be normalized to either total number of reads [78–80] or 16S rRNA gene copies [81–85]. Although the 16S rRNA gene is frequently used for normalization, it may not always yield accurate estimation of ARGs as multiple copies of this gene can be present within a genome [86]. Recently, it has been shown that using multiple single-copy marker genes [87] or comparison of each gene count with the whole content of ARGs [88] may be an alternative approach for data normalization. In summary, the methods for data normalization should be taken into consideration when comparing the resistome in the digestive tract of food-producing animals across different studies.

#### Identification of microbial host of ARGs using metagenomic binning

Metagenomic binning enables near-complete microbial genomes to be reconstructed from metagenomic sequencing data, which has been extensively used in the food-producing animals such as swine [89, 90], chickens [91, 92], and ruminants [93–95]. Technically, binning of assembled metagenomic sequences requires binners such as MaxBin [96, 97], MetaBAT [98, 99], or Concoct [100]. The obtained metagenomic-assembled genomes (MAGs) can be further refined using tools such as DASTool [101] to remove non-microbial DNA and increase predictive accuracy. The completeness and residual DNA contamination of MAGs can be calculated using CheckM v1.0.6 [102] based on lineage-specific conserved marker gene sets in each genome. Finally, the classification of MAGs can be achieved by blasting them against the reference genomic database. A recent review comprehensively evaluated the performance of 15 genome binning tools and suggested that Groopm2 [103] achieved the highest purity while MetaBat2 had higher completeness than other binners using metagenomic datasets generated from the contents of the chicken gut [104]. In recent years, metagenomic binning analysis has been applied in combination with resistome analysis to identify the microbial host of ARGs in environmental samples from public/private houses [105], lake sediments [106], and a wastewater treatment plant [107]. However, the use of binning analysis is still very limited for resistome analysis of samples collected from human, environment, and food-producing animals. One potential reason could be that binning analysis requires either genomic sequences derived from uncharacterized microorganisms (reference-dependent) or substantial computing resources (reference-independent) [108]. With the expansion in the reference database of various microorganisms as well as development of less computationally-intensive tools (e.g. MetaBMF [109]), we speculate that binning analysis will be widely used to identify microbial hosts of ARGs in the digestive tract of food-producing animals in the future.

#### Factors affecting the accuracy of resistome analysis

In addition to the difference in analysis approach (read- and assembly-based), reference databases (ARDB, CARD), as well as data normalization method, other factors may also affect the predictability of the resistome. First of all, there are currently at least 50 tools/pipelines that can be used to analyze the resistome [110, 111]. Since the features (e.g. read-based or assembly-based) of these analysis tools differ greatly, it is necessary to consider benchmarking of resistomes that are analyzed using different pipelines [112]. Secondly, the composition of resistome is heavily influenced by the phylogenetic profile of the bacterial population. Considering that there are still a large number of bacteria that possess unidentified ARGs, the known ARGs may only represent a small part of the true resistome. It is reasonable to assume that with future advances in sequencing as well as the expansion in ARGs and phylogenetic databases, new ARGs will be identified and characterized. In addition, sequencing depth can also affect the profile of resistomes. For example, by sampling three potential environmental ARG reservoirs (pig caeca, river sediment, effluent) and sequencing them using shotgun metagenomics, Gweon et al. [113] found that at least 80 million reads per sample were required to cover the full richness of different ARG families within these environments. In addition, Zaheer et al. [114] showed that the number of reads being assigned to ARGs increased significantly with increasing sequencing depth (from 26 to 117 million reads), with a depth of approximately 60 million being suitable to describe the resistome in feces from beef cattle. While the optimal sequencing depth needed for resistome analysis in the digestive tract of food-producing animals still deserves investigation, these studies suggest that a balance between sequencing depth and cost needs to be defined in order to obtain reasonable results.

### Profiles of resistome in the digestive tract of food-producing animals

Recently, a number of studies characterizing the resistome in food-producing animals been conducted as a result of a reduction in the cost of metagenomic sequencing as well as the development of pipelines and resources for resistome analysis [75, 115]. Here we summarize the recent advances in the analysis of resistome in the digestive tract of ruminants, swine, and poultry, focusing on the fecal resistome. Feces often represent the principal conduit of ARG contact from livestock with soil, water, vegetation and humans. This is particular true for less developed countries where food-producing animals are often raised in close proximity to human habitations [116].

#### Resistome in the digestive tract of swine

Antimicrobials belonging to penicillin, tetracycline, and macrolide groups are commonly used in swine [117, 118]. In general, oral administration via water or feed is the most common route of antimicrobial administration in swine [119], for treatment of infectious disease, metaphylaxis, prophylaxis and growth promotion [118]. Here we summarized findings from 13 studies investigating the resistome in the digestive tract of swine (Table 1). Most of these studies investigated the effect of anti-microbial use (AMU) [120–129] on the profiles of resistome in the swine digestive tract, while only one study reported the resistome profile in the absence of AMU [130]. Two studies failed to mention if antimicrobials were used or not [30, 131].

**Table 1:** Resistome in the digestive tract of swine based on metagenome sequencing

Sample	Use of antimicrobials	Name of antimicrobial	Major findings	Reference
Feces (*n* = 6)	NM	–	–Predominant by tetracycline, followed by MLS, aminoglycoside, and β-lactam.	[30]
Feces (*n* = 6)	Yes	NS	–For both 1- and 8-month-old pigs, tracycline was the most abundant ARG, followed by aminoglycoside MLS. –The abundances of bleomycin, fosmidomycin, and polymyxin decreased over age (8- vs. 1-month-old)	[120, 121]
Feces (*n* = 181)	Yes	NS	–Predominant by tetracycline, followed by macrolide in all 9 countries. –Countries with similarly high (such as Spain and Italy) or low (Denmark and the Netherlands) usage of antimicrobials have similar resistome profiles	[122]
Feces (*n* = 25)	Yes	NS	–Positive associations between use of antimicrobials and ARG for macrolides and tetracyclines, but not for β-lactams classes.	[123]
Feces (*n* = 6)	Yes	Oxytetracycline	–127 ARGs related to 19 classes were identified. –41 ARGs, mainly from the tetracycline, β-lactam and MDR classes were enriched after administration.	[124]
Feces (*n* = 24)	Yes	Tulathromycin	–The abundance of fecal ARGs in piglets changed over time. –Perinatal use of tulathromycin had no effect on the abundance of ARGs in piglets.	[125]
Feces (*n* = 26)	Yes	NS	–Predominant by tetracycline, followed by MLS.	[126]
Feces (*n* = 4)	Yes	NS	–Predominant by tetracycline, followed by aminoglycoside, and MDR.	[127]
Feces (*n* = 38)	Yes	NS	–Predominant by tetracycline, followed by aminoglycosides, MLS, and oxazolidinones.	[128]
Ileum (*n* = 23) and colon content (*n* = 24)	Yes	Chlortetracycline and virginiamycin	–No significant difference in the structure and diversity of ARGs and MGE after administration of low-dose antimicrobials. –Predominant by tetracycline, followed by macrolide, aminoglycoside, lincosamide, and streptogramin in colon. –Predominant by tetracycline, followed by penam, fluoroquinolone, aminoglycoside, and cephalosporin in ileum. –No difference in structure and diversity of ARGs and MGE after administration for both samples.	[129]
Feces (*n* = 16)	No	–	–Predominant by tetracycline, followed by MLS, aminoglycoside and β-lactam.	[130]
Feces (*n* = 36)	NM	–	–Predominant by tetracycline, with *tetQ*, *tetW*, *tetO*, *tet32*, and *tet44* being the most abundant.	[131]

*ARG* antimicrobial resistant gene, *MGE* mobille genomic element, *MLS* macrolide-lincosamide-streptogramin, *MDR* multidrug resistance, *NM* not mentioned, *NS* not specified.

Joyce et al. [130] investigated the resistomes of swine feces and found that the core resistome (present in all samples) contained 56 ARGs with five tetracycline resistant genes (*tetW*, *tetQ*, *tet44*, *tet37*, *tet40*) being the most abundant, and the accessory resistome (detected in at least one sample but not present in all samples) being comprised of 201 ARGs. This finding provides insights into the profiles of fecal resistome in swine in the absence of antimicrobial selective pressure and emphasizes that AMU is not the only factor that dictates the nature of the resistome within the digestive tract of swine.

Resistome profiles have also been shown to differ among AMU protocols. For example, a study compared the profiles of resistome in feces of pigs of differing age and found that tetracycline was the most abundant ARG for both 1- and 8-month-old pigs, followed by aminoglycoside and macrolide-lincosamide-streptogramin (MLS) [120]. These authors further found that the abundances of several classes of ARGs in feces including bleomycin, fosmidomycin, and polymyxin decreased with age (8- vs. 1-month-old), possibly as a result of the reduction in the use of these antimicrobials in older swine [120]. However, it should be noted that only 3 samples of each age were analyzed in that study, which may lead to low reproducibility of the results. In addition, Van Gompel et al. [123] found a positive association between the use of macrolides and tetracyclines and AMR in pigs raised in 9 European countries. However, they didn’t find significant associations between the high use of β-lactams and the abundance of their resistance genes in the feces of younger pigs. These authors also reported that the profiles of fecal resistome in pigs were country-specific [122], possibly a reflection of differences in AMU (frequency/dosage) among countries with it being high in Spain and Italy and low use in Denmark and the Netherlands. Administration of oxytetracycline significantly enriched the abundances of 41 ARGs in swine feces, mainly members of the tetracycline, β-lactam and multidrug (MDR) classes, with an increase in *Escherichia* and *Prevotella* within the bacterial population as compared to non-medicated pigs [124]. It was proposed that these ARG-carrying bacteria may have the potential to transfer ARGs to other susceptible bacteria in the digestive tract of swine [124]. One limitation of this study is that dietary factors, which may have a significant impact on microbiota and associated resistome, were not reported. In addition, a study investigated the effect of perinatal use of tulathromycin on the profiles of fecal resistome in pre-weaned piglets [125]. Although these authors identified a total of 127 ARGs related to 19 different classes, perinatal use of antimicrobials had no impact on the structure of fecal microbiota or the abundance of ARGs in feces from pre-weaned piglets [125]. These inconsistent findings suggest that AMU may not be the only factor that impacts the resistome profiles in the digestive tract of swine, and that other factors such as diet, growth stage, housing type and other environmental influences also play a role.

In addition to feces, the resistome in the intestinal contents of weaned piglets has been recently reported [129]. These authors reported that the resistome in the colon contained ARGs mainly associated with tetracycline, MLS, and aminoglycoside resistance. In the ileum, tetracycline was also the predominant ARG, while penem, fluoroquinolone, aminoglycoside, and cephalosporin ARGs also accounted for a large proportion of resistome. In addition, they found that administration of low-dosage antibiotics for 4 weeks had no significant impact on the structure, diversity, or diversity of resistome in either ileum or colon contents. Considering that only one sampling was available in this study, the long-term effect of exposure to antibiotics on resistome needs further investigation. Nevertheless, it should be noted that the microbiota in feces may not fully represent the microbiota within the whole gastrointestinal tract [132]). More research is needed to investigate the profiles of resistome in the various regions of the swine gastrointestinal tract in addition to that in feces so as to have a comprehensive understanding of the resistome throughout the digestive tract of swine.

#### Resistome in the digestive tract of chickens

In intensive poultry farming, antimicrobials such as tetracycline, bacitracin, tylosin, salinomycin, virginiamycin and bambermycin are often used [117, 133]. In US, tetracyclines represent more than two-thirds of antimicrobials administered in poultry production [134], while they represent only 37% [135] in the EU poultry industry. Amoxycillin, oxytetracycline and ceftriaxone are the most commonly used antimicrobials in poultry, followed by ofloxacin and norfloxacin in China [136]. Inclusion in feed is the most common route of administration of antimicrobials in poultry, mainly to prevent necrotic enteritis caused by *Clostridium perfringens* and coccidiosis [137].

Studies of the resistome in the digestive tract of chickens are relatively limited as compared to swine (Table 2). As with swine, it has been reported that tetracycline, aminoglycoside, and MLS are the most abundant ARG classes in chicken feces (20-day and 80-day old) [120, 121], despite that only 2 samples were collected from each age group. Subsequent studies confirmed that ARGs belonging to these classes were predominant, although one study reported MDR as the predominant ARG class in chicken feces [125, 137]. A total of 49 core ARGs were identified in 178 fecal samples collected from 9 European countries, with tetracycline and macrolide ARGs accounting for the majority of the resistome [121]. Although these authors reported a more diverse resistome in fecal samples from chickens than pigs [121], dietary compositions of chickens raised in different countries were unavailable, making it difficult to determine whether dietary factors may contribute to the diversity of the resistome in chickens. A recent study in China compared the fecal resistome of chickens at poultry farms to those at live poultry markets in China [139]. For both sites, ARGs conferring resistance to aminoglycoside, tetracycline, MLS, and β-lactam were more abundant than those associated with other classes. In addition, these ARG classes were more abundant in birds at the poultry market than in those on farm [139], an observation that could reflect changes in microbiome due to increased stress in birds that are marketed through live poultry trade.

**Table 2:** Resistome in the digestive tract of poultry based on metagenome sequencing

Sample	Use of antimicrobials	Name of antimicrobial	Major findings	Reference
Feces (*n* = 6)	NM	–	–Predominant by tetracycline, MLS, aminoglycoside, and β-lactam.	[30]
Feces (*n* = 4)	Yes	NS	–Predominant by tetracycline, followed by aminoglycoside.	[120, 121]
Feces (*n* = 178)	Yes	NS	–Tetracycline, macrolide, β-lactam and aminoglycoside AMR made up the majority of ARGs.	[122]
Feces (*n* = 12)	Yes	Chlortetracycline	–Predominant by MDR, followed by aminoglycoside, and tetracycline. –Chlortetracycline at low or therapeutic doses did not alter the relative abundance of total ARGs and predominant ARG classes.	[126, 138]
Feces (*n* = 63)	Yes	NS	–Predominant by tetracycline, followed by MLS, aminoglycoside, and β-lactam. –More abundant ARGs in the fecal samples collected in markets than farms.	[139]
Feces (*n* = 15)	Yes	Ampicillin	–Predominant by tetracycline. –Ampicillin led to the increase in the abundance of ARGs belonging to β-lactam and bacitracin, and decrease of those belonging to tetracycline. –Increase in β-lactam, bacitracin-resistance, and MDR genes were more evident for oral than intramuscular administration of ampicillin.	[140]
Cecum (*n* = 10)	Yes	NS	–Predominant by tetracycline, MLS, and cephalosporin resistant genes are the most abundant in two altitudes (730 m and 3300 m). –Differential abundant MLS, cephalosporin, and tetracycline between low and high altitudes.	[141]

*ARG* antimicrobial resistant gene, *MLS* macrolide-lincosamide-streptogramin, *MDR* multidrug resistance, *NM* not mentioned, *NS* not specified.

A few studies also investigated the use of specific antimicrobials on the profiles of the fecal resistome in poultry. For example, administration of ampicillin led to an increase in the abundance of most β-lactam and bacitracin ARGs and a decrease in ARGs associated with tetracycline classes in chicken feces [140]. In addition, this increase in β-lactam, bacitracin-resistance, and MDR ARGs were more evident if the ampicillin was administered orally as compared to intramuscularly [140]. However, therapeutic dosages of chlortetracycline in feed increased the abundance of tetracycline resistance (*tetA* and *tetW*) and reduced multiple MDR ARGs in broiler feces [138]. This response to chlortetracycline was attributed to a decline in the population of *Escherichia*, a major host of MDR ARGs, and the enrichment of *Bifidobacterium,* which harbours more *tetW* [138]. These findings indicate that dosage, method of administration and type of antimicrobial all influence the profile of the resistome within the digestive tract of poultry. In addition to the fecal resistome, a study compared the profile of cecum resistome in chickens housed at low (730 m) and high (3,300 m) altitudes [141]. While tetracycline, MLS, and cephalosporin were the predominant classes of ARGs identified at both altitudes, these classes were more abundant in chickens reared at low than high altitude [141], This may reflect the lower abundance of ARGs in soil and water bacteria at high-altitude environments that could be transferred to chickens, but such a hypothesis would require further investigation.

#### Resistome in the digestive tract of ruminants

At least ten classes of antimicrobials are used in ruminants (e.g. tetracyclines, amphenicols, penicilins, cephalosporins), which are mainly used to prevent or treat diarrhoea, respiratory disease, navel infections, liver abscesses, foot rot and joint and uterine infections in beef and dairy cattle [142]. In dairy cows, penicillins, cephalosporins, or other beta-lactams are used to prevent and control mastitis [143]. In addition, acute puerperal metritis can be treated with penicillin/ampicillin in conjunction with oxytetracycline or ampicillin and cloxacillin [144]. The route of administration depends on the type of antimicrobial. For example, in dairy cows, penicillins, cephalosporins, or other beta-lactams are often infused into the mammary gland, whereas penicillins, macrolides, aminoglycosides, and fluoroquinolones are administered parenterally, while sulfonamides [143–145] are administered orally. For beef cattle, tylosin, chlortetracycline, oxytetracycline, virginiamycin, are used for liver abscess prevention, while macrolides and tetracycline’s are often administered to prevent or treat bovine respiratory disease [146]. In sheep and goats, penicillin is often used due to its low cost and the low risk associated with off-label use, as few antimicrobials are specifically registered for use in small ruminants [147–149].

#### Rumen resistome

The rumen harbors a dense microbiota including bacteria, methanogens, protozoa, fungi, and phages [150]. Consequently, the resistome of this unique microbial ecosystem has been investigated in several studies [151, 152]. Recent research showed that rumen microbiota in cattle and sheep also harbor a vast reservoir of ARGs, with the abundance and gene type affected by factors such as diet and/or use of antimicrobials (Table 3). For example, Hitch et al. [153] identified 30 ARGs in the rumen of sheep that harboured a high abundance of daptomycin and colistin resistance genes, both of which represent ‘last-resort’ antimicrobials against gram-positive bacterial infections in humans [166, 167]. Although not specifically documented, there is a risk that these ARGs could be transferred to human-associated bacteria through close contact on farm, in abattoirs or consumption of contaminated meat or milk products. In addition, Auffret et al. [152] comparatively investigated the effects of diet (concentrate:forage ratio) and breed on the profiles of resistome in the rumen of beef cattle. These authors found ARGs belonging to 13 ARGs classes with macrolide, chloramphenicol, β-lactam, and aminoglycoside classes predominating. Chloramphenicol and aminoglycosides ARGs were predominant in rumen samples of beef cattle fed either high forage or high concentrate diets. However, breed had no significant effect on the profile of ARGs. This finding suggests that diet may have a more significant impact on the rumen resistome than the genetics of the host.

**Table 3:** Resistome in the digestive tract of ruminants based on metagenome sequencing

Site	Animal	Use of antimicrobials	Name of antimicrobial	Major findings	Reference
Rumen	Dairy cattle (*n* = 49)	No	–	–Predominant by tetracycline class. –Abundance of resistome could be linked to milk protein yield.	[25]
Rumen	Beef cattle (*n* = 50)	No	–	–Higher diversity and abundance in high concentrate diet. –Chloramphenicol, microcin are predominant in high forage diet. –Aminoglycoside, streptomycin are predominant in high concentrate diet. –No breed effect on resistome.	[152]
Rumen	Beef cattle (*n* = 10)	Yes	Monensin and tylosin	– predominant by tetracycline and MLS. –No effect of antimicrobials on resistome.	[151]
Rumen	Sheep (*n* = 10)	NM	–	–Daptomycin and colistin are present in all samples.	[153]
Feces	Dairy calf (*n* = 12)	No	–	–329 ARGs conferring resistance to 17 classes of ARG. –The abundance of ARGs declines during nursing.	[85]
Feces	Beef cattle (*n* = 8)	Yes	NS	–Predominant by tetracyclines, macrolides, aminoglycoside. –The number of reads being assigned to ARGs, but not the relative proportions of ARGs, increased with sequencing depth.	[114]
Feces	Beef cattle (*n* = 14)	Yes	NS	–Trimethoprim and aminoglycoside classes were only identified in calf feces, while tetracycline major facilitator superfamily (MFS) alignments only in adult cattle feces. –More abundant macrolide efflux pumps and lincosamide nucleotidyltransferases in adult cattle feces.	[154]
Feces	Beef cattle (*n* = 16)	Yes	NS	–Predominant by tetracycline and MLS classes. –Diversity of resistome decreased over time. –AMR were not identified in beef products.	[155]
Feces	Beef cattle and dairy cattle (*n* = 8)	Yes	NS	–Feces had the greatest number of ARGs in conventional system. –More tetracycline, macrolide, and aminoglycoside in conventional system. –Tetracycline and MLS classes are more abundant in feedlot cattle than in dairy cow. –β-lactam class is more abundant in dairy cow feces.	[156]
Feces	Dairy cattle (*n* = 6)	Yes	–	–Predominant by tetracycline class. –Ceftiofur enriched ARGs belonging to β-lactam class.	[157]
Feces	Beef cattle (*n* = 16)	Yes	Ceftiofur and Chlortetracyclin	–Ceftiofur was not associated with changes to β-lactam resistance genes. –Chlortetracycline increased relative abundance of tetracycline resistance genes.	[158]
Feces	Beef cattle (*n* = 6)	Yes	NS	–Predominant by tetracycline, MLS, β-lactam, and aminoglycoside. –No difference in the profiles of resistome between two systems.	[159]
Feces	Beef cattle (*n* = 16)	Yes	Tylosin	–No effect of tylosin on the abundance of resistome. –Predominant by tetracycline, MLS, and elfamycin.	[160]
Feces	Beef cattle (*n* = 30)	Yes	Tulathromycin	–No effect of antimicrobials on resistome.	[161]
Feces	Veal calf (*n* = 42)	Yes	Oxytetracycline	–Sub-therapeutic administration of oxytetracycline do not result in increased *tetM* resistance levels as observed in the therapeutic group.	[162]
Feces	Veal calf (*n* = 24)	NM	–	–Predominant by tetracyclines, aminoglycosides and MLS.	[163]
Feces	Beef cattle (*n* = 28)	No	–	–Tetracycline (62.3%) and macrolide (25.6%) classes are predominant. –*S*. *cerevisiae* fermentation product did not impact resistome.	[164]
Feces	Beef cattle (*n* = 12)	Yes	NS	–Predominant by tetracycline and macrolide.	[165]

*ARG* antimicrobial resistant gene, *MLS* macrolide-lincosamide-streptogramin, *MDR* multidrug resistance, *NM* not mentioned, *NS* not specified.

Two recent studies showed that the prevalence of AMR may not be necessarily associated with the use of antimicrobials in rumen of beef and dariy cattle. Thomas et al. [151] detected ARGs belonging to the macrolide (*ermF* and *ermG*) class in the rumen of beef cattle supplemented with monensin and tylosin as well as those that did not receive antimicrobials. As the period of supplementation of antimicrobials only lasted for 74 days, the resistome profile may change with longer term supplementation. More recently, Xue et al. [25] identified ARGs belonging to 26 classes of ARGs using samples collected from 49 dairy cows that did not receive antimicrobials during the experiment. They found that ARGs encoding resistance to tetracycline were the most common, followed by those encoding glycopeptide and fluoroquinolone resistance. One possible reason for this finding could be that these animals were exposed to antimicrobials shortly after birth (e.g. disease prevention or treatment), which could have had a long-term impact on the rumen resistome. As the number of related studies are very scarce, more experiments should be conducted to investigate the effect of use of antimicrobials on the profiles of the rumen resistome in ruminants such as cattle, sheep, and goats.

In addition to these metagenome-based *in vivo* studies, a microbial-genomics-based *in vitro* study analyzed 435 genomes of ruminal bacteria and archaea and identified a high abundance of genes encoding tetracycline resistance using a variety of ARGs databases (e.g. ResFinder, Resfams, ARG-ANNOT), a finding which is consistent with previous *in vivo* studies [168]. However, the prevalence of genes resistant to ARG classes can be influenced by the nature of the analytical approach employed. For example, the prevalence of beta-lactam and vancomycin resistant genes was only detected using Resfams, but not ResFinder or ARG-ANNOT, demonstrating that variable tools for resistome analysis may generate different results. Consequently, results that are generated with different computational pipelines need to be interpreted with caution. By aligning several genes conferring resistance to aminoglycosides, beta-lactams, macrolides, tetracyclines, as well as vancomycin to selected rumen metatranscriptomic datasets, the authors confirmed the expression of additional ARGs including *tetQ*, *tetW*, *tetO*, and *tet37*. While these findings need validation *in vivo*, our recent study found that *tetW* and *tetQ* were the most abundant ARG transcripts (expressed resistome) in the rumen of beef cattle (Ma et al, submitted). The expression of these tetracycline resistant genes and their functions need further investigation.

#### Fecal resistome

Tetracycline is the most abundant ARG class in feces of ruminants, and MLS along with aminoglycoside ARGs have been also reported to account for a large proportion of the fecal resistome of ruminants (Table 3). In general, the diversity of the fecal resistome decreases with increasing age in cattle [154]. While trimethoprim and aminoglycoside classes were only identified in feces from calves, tetracycline superfamily alignments were only identified in mature cattle, with macrolide efflux pumps and lincosamide nucleotidyltransferases also more common in adult cattle than calves [155].

Numerous studies have evaluated the effect of the use of antimicrobials on the profiles of the fecal resistome in cattle. In general, more abundant tetracycline, macrolide, and aminoglycoside ARGs were detected in feedlot cattle administered antimicrobials in feed as compared to those that were not [156]. Specifically, the use of ceftiofur, a 3rd-generation cephalosporin for the treatment of respiratory disease and foot rot [169], enriched for β-lactam ARGs [157, 158]. Similarly, cattle fed chlortetracycline showed a significant increase in the relative abundance of tetracycline ARGs in feces [158]. Nevertheless, some studies found no difference in the profiles or abundance of fecal resistome in feedlot cattle under conventional vs. raised without antimicrobials production conditions [159]. For example, supplementation of tylosin, a macrolide that inhibits protein synthesis in bacteria [170], did not affect the abundance of these ARGs within resistome of feces from beef cattle [160]. Moreover, no difference was observed in the profiles of fecal resistome of cattle injected with or without tulathromycin, a macrolide used to prevent and treat bovine respiratory disease [171, 172]. This was in spite of the composition of both the microbiome and resistome changing over 11 days post-injection [161]. The difference in these findings may be attributed to the dosage of antimicrobial used, as administration of low-dosage of oxytetracycline (100–200 μg per day) in veal calves did not result in increased *tetM* resistance levels as was observed in high-dosage (1 g per day) group after 42 days of administration [162]. Based on these findings, administration of antimicrobials may not necessarily impact the profiles of resistome in fecal microbiota of ruminants. However, the transmission of ARGs from fecal to environmental microbiota (e.g. soil and water) in ruminants administered antimicrobials needs further investigation.

### Future focus on resisome analysis in the digestive tract of food-producing animals

**Fig. 2:**
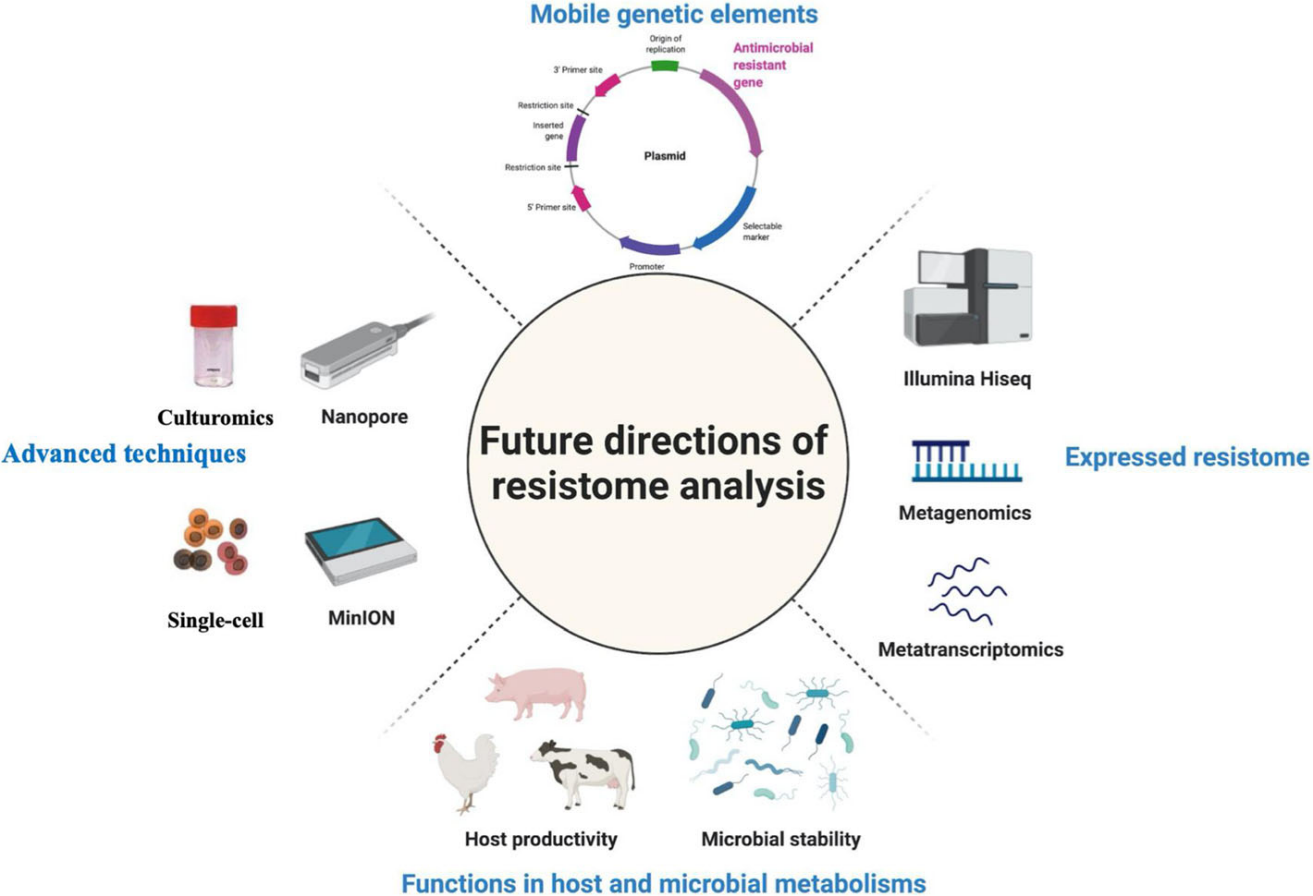
Future directions of resistome analysis in the digestive tract of food-producing animals (Created with BioRender.com)

Although some progress has been made, our current knowledge of resistome in the digestive tract of food-producing animals is still limited and more efforts are warranted. Here we propose several potential approaches that could be used to advance our understanding of the resistome within food-producing animals (Fig. 2).

#### Application of long-read sequencing techniques

While short-read sequencers such as Illumina’s HiSeq and MiSeq [173] produce reads of up to 600 bases, long-read sequencing technologies, featured by Pacific Biosciences single-molecule real-time sequencing and Oxford Nanopore Technologies’ nanopore sequencing, routinely generate reads more than 10 kb [174]. Compared with short-read sequencing, long-read sequencing improves *de novo* assembly, mapping certainty, transcript isoform identification, and detection of structural variants [175]. Moreover, it is difficult to determine the exact genomic context of ARGs using short-read sequencing, while long read sequencing offers a solution to this problem as repeat regions can be spanned and defined [176]. Attempts have been made to use long read sequencing to characterize resistome associated with foods [177], stormwater [178], feces and preterm babies [179]. Along with resistome mapping tools specifically developed for long-read sequencing such as poreFUME [180] and ARGpore [181], the results of the above studies showed that long-read sequencing can be used to establish the context of ARGs within the resistome. Additionally, the combination of long-read (Oxford Nanopore) and short-read (Illumina) metagenomic sequencing has been proven to be a promising approach to comprehensively investigate the resistome in environmental samples such as sewage sludge [182] or in bacterial isolates [176]. Long-read sequencing looks particularly promising for further characterizing factors that regulate the expression of ARGs and the role mobile genetic elements play in their dispersion among bacterial members within microbiomes [183]. With improvements in error correction and continued reductions in sequencing cost, we foresee long-read sequencing as a promising approach to further investigating the resistome profiles in the digestive tract of food-producing animals.

#### Application of single-cell sequencing techniques

Single-cell sequencing refers to the sequencing the genome or transcriptome of a single cell to characterize the genomic, transcriptomic and metabolomic function of single cells [184]. This is accomplished by the physical separation of single cells from environmental samples, followed by sequencing and assembly of their individual genomes which are then subject to a series of downstream analysis. Single-cell sequencing has proven to be a powerful tool for studying unculturable organisms and delineating complex populations since its first inception in 2005 [185]. However, for a long time, the widespread use of single-cell sequencing was limited due to its high cost. With the continuous efforts made in lowering detection costs, single-cell sequencing is increasingly used in various fields such as cancer research, immunology, and microbiology [186]. The bioinformatics pipelines of single-cell RNA sequencing technologies have been recently reviewed [187]. Lan et al. performed high-throughput single-cell genome sequencing (SiC-seq) on synthetic communities to analyze the distribution of ARGs, virulence factors and phage sequences in environmental microbial communities [188]. The work showed that single-cell sequencing technologies can play an important role in the identification of antimicrobial resistant microorganisms.

#### Application of culturomic techniques

Culturomics refer to a culture-dependent approach to study complex microbial ecosystems such as the human and animal intestinal tract. This approach can be used to complement metagenomics by overcoming the depth bias inherent in metagenomic sequencing [189]. Culturomics use high-throughput tools such as matrix-assisted laser desorption ionization time-of-Flight (MALDI-TOF) mass spectrometry to comprehensively identify bacteria from environmental samples to the strain level [190]. McLain et al. summarized the use of culturomics for identification of AMR in agroecosystems and suggested that culture as well as isolation of individual microbes carrying ARGs are essential for determining multi-antimicrobial resistance phenotypes [191]. A recent review by Bilen et al. [192] updated the inventory of prokaryotes (from 2,172 to 2,776 species) from different human body sites that was originally published by Hugon et al. [193]. In this update, culturomics contributed up to 66.2% of the updates to this repertoire. This review concluded that culturomics proved useful in identifying new ARG-carrying bacteria. Despite these efforts, the use of culturomics to detect AMR in the digestive tract of food-producing animals has not been reported. We therefore speculate that culturomics could be an ideal complementary approach to metagenomics sequencing to study the resistome in the digestive tract of food-producing animals.

#### Identification of resistome on mobile genetic element

An important route for bacteria to acquire antimicrobial resistance is through horizontal gene transfer (HGT) [194–196], mediated by mobile genetic elements (MGE) such as plasmids [197]. It has been shown that plasmids are one of the MGEs that are abundantly present in bovine rumen bacterial populations [198]. In addition, plasmid-mediated resistance genes such as *qnrA*, *blaCTX-M* and *mcr-1* have been identified within the resistome of swine [199]. Although metagenomic approaches have been used to characterize plasmid associated ARGs in activated sludge [200, 201] and the human gut [202], the profiles of plasmid-associated ARGs have not been extensively examined in food-producing animals. Our recent study showed that a total of 90 ARGs belonging to 15 classes were plasmid-associated in the rumen of beef cattle, with tetracycline, aminoglycoside, and MLS being the ARGs most often associated with MGE’s (Ma et al., submitted). With the continuous curation of reference databases such as a CLAssification of Mobile genetic Elements (ACLAME) [203]), PlasmidFinder [204], and MOB-suite [205], there is a need to characterize the role of plasmid-associated ARGs within the reistome within the digestive tract of food-producing animals.

#### Investigation into the expressions of resistome

Current studies of the resistome in food-producing animals are mainly based on technologies such as metagenomic sequencing. However, expression of ARGs within the resistome and the factors that influence it are still largely unclear in food-producing animals. Recent studies have used metagenomics and metatranscriptomics to assess the resistome of wastewater treatment plants and revealed that the location of the plant not only affects the types of ARGs present, but also their expression [206]. Expression of ARGs in the fecal resistome of chickens and pigs has been examined, but only in a limited number of samples (*n* = 6) for each species [30]. Our recent study showed that the expressed ARGs in the rumen of beef cattle only represented less than 1% of the resistome, with *tetW* and *mefA* exhibiting the highest level of expression (Ma et al., submitted). Moreover, we found significant differences in the abundance of microbial ARGs (metagenomic profiling) but not expressed ARGs (metatranscriptomic profiling) in the rumen in different beef breeds. In this regard, more studies based on metatranscriptomics are needed to understand the expression of the resistome in the digestive tract of food-producing animals. Although numerous tools have been developed for resistome analysis, they have been designed for metagenomic datasets and their suitability for metatranscriptomic datasets needs further validation.

#### Functions of the resistome in addition to AMR

Other than transmission of AMR, studies have shown that the ARGs may also have other functions that impact both host and the microbiome within the digestive tract of food-producing animals. For example, some AMR elements such as efflux pumps may regulate amino acid, fatty acid or nucleotide metabolism of microbiota while also conferring intrinsic antimicrobial resistance [207–209]. A study based on metagenomic sequencing also revealed that dairy cattle managed in the same manner and fed the same diet, but with high and low milk protein yields exhibited different rumen resistome profiles (resistotypes) [25]. Specifically, these authors reported that the abundance of 128 ARGs differed in dairy cows with high and low milk protein yield. In particular, cows with low milk protein yield had a higher abundance of *mfd* (encoding a transcription-repair coupling factor involved in strand-specific DNA repair [210]) and *sav1866* (encoding a multidrug export ATP-binding/permease protein [211]). While this study revealed a potential relationship between ARGs and host production, a recent study by our group found a positive correlation between the abundance of multiple subtypes of expressed ARGs (e.g. *tetW* and *tetQ*) and various metabolism pathways within the active rumen microbiome (Ma et al., submitted). In addition, we found that higher expression of *tetW* may be associated with increased stability of the active rumen microbiota, which may be more resistant to external perturbations such as the administration of antimicrobials in feed. However, as the above studies are based on correlation, they may not reflect causation, and the exact roles ARGs in maintaining or regulating host/microbial functions needs further elucidation. A future goal could be to differentiate those ARGs that may play an ancillary role in microbiome functions that influence host productivity from those that specifically result in the transmission of AMR.

### Limiting the use of antimicrobials to control host, microbial, and environmental transmission of ARGs

**Fig. 3:**
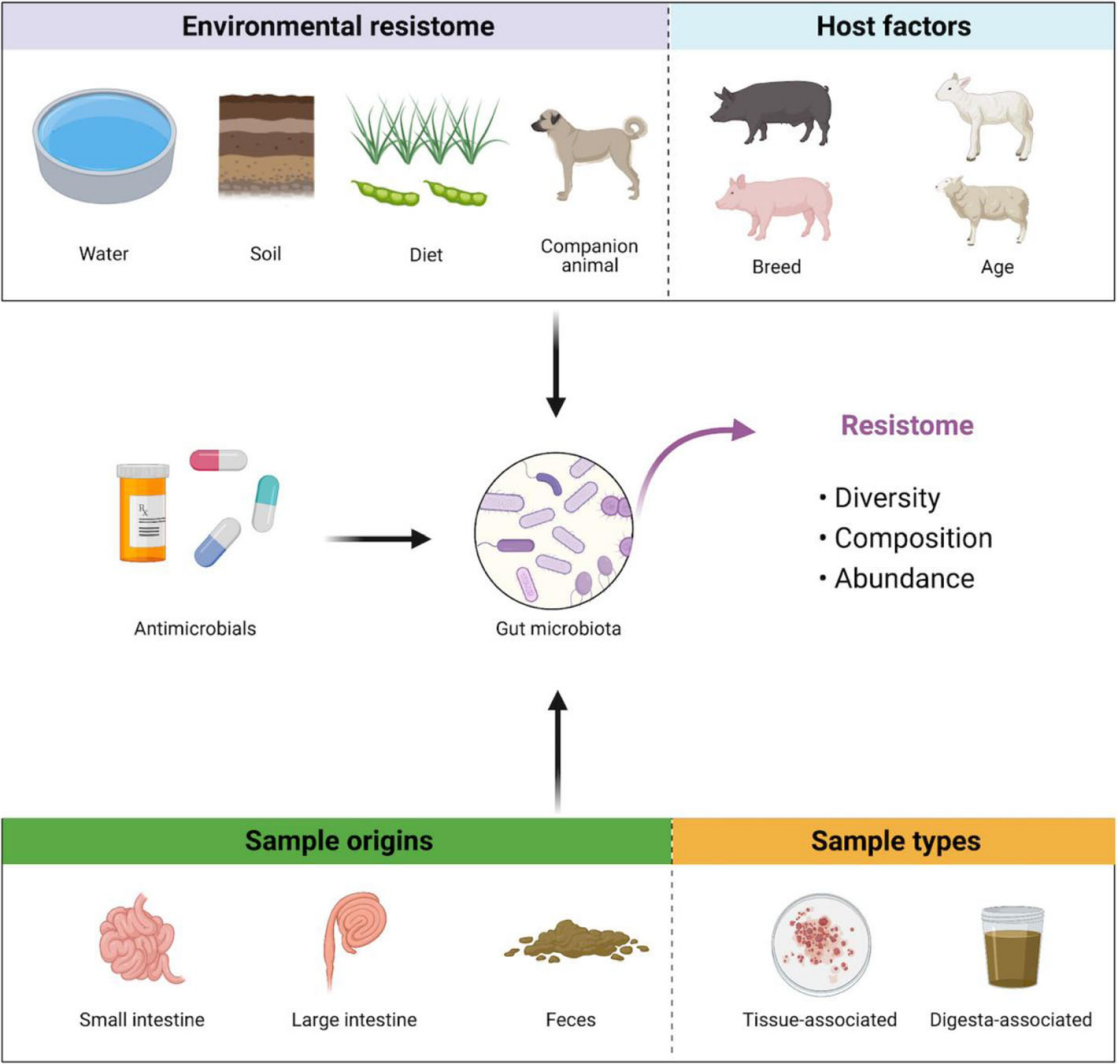
Host (breed and age), microbial (sample origin and type), and environmental (water, soil, diet, and companion animal) factors that may impact the effect of administration of antimicrobials on the diversity, composition, and abundance of resistome in the digestive tract of food-producing animal (Created with BioRender.com)

Efforts have been made to minimize or limit the spread of AMR by reducing AMU in food producing animals. Indeed, several of the above-mentioned studies showed a reduction in the diversity or abundance of ARGs after eliminating AMU in food-producing animals. However, other studies also found no difference between the resistome profiles of food-processing animals that received or did not receive antimicrobials. We propose several factors that may impact resistome as a result of the use of antimicrobials in food-producing animals (Fig. 3). First, several recent studies showed that the gut microbiome is heavily influenced by the host in cattle [212–214], swine [215] and poultry [216, 217]. This raises the possibility that the host genetics may also affect the profiles of microbial-associated resistome in the digestive tract of livestock and poultry. Although Auffret et al. [152] did not find significant impact of cattle breed on the fecal resistome, our recent research found that crossbred cattle had a less diverse resistome as compared to purebred cattle (Ma et al., submitted). In addition to breed, age may also influence the impact of antimicrobials on the resistome, as the gut microbiota may be less resilient to external perturbations such as the use of antimicrobials in early life, with potential life-long impacts on the gut microbiome [218, 219]. We thus speculate that the profiles of microbial-associated resistome may also be more likely to change in response to the use of antimicrobials at an early age in food-producing animals.

### Companion animals as a source of ARGs in food animal production systems

In addition, it is noticeable that ARGs can also be transmitted between companion animals and food production animals and humans. Companion animals such as dogs and cats are present on most farms [220], making the transmission of ARGs possible among them and food-producing animals as well as human beings. According to a recent review, β-lactams such as amoxicillin are the most commonly used antimicrobials for dogs and cats in European countries including UK, Italy, and four Nordic countries [221]. A recent study investigated the profiles of resistome in the digestive tract of dogs and cats and found that tetracycline and aminoglycoside resistance genes were the most abundant among the 23 classes of ARGs, indicating that the gut microbiota of dogs and cats is also a reservoir of ARGs [222]. We thus speculate that there could be many shared ARGs between these companion and food-producing animals living together in farms considering the similarity of the profiles of predominant ARGs (e.g. *tetW* and *tetQ*) in their digestive tract. In horses, antimicrobials (e.g. penicillins) are used mainly to treat equine skin infections [223] and colitis/diarrhea [224]. However, studies of the resistome in the digestive-tract of horses are still limited. A study showed that prophylactic administration of a macrolide antimicrobial with rifampin promoted MDR genes in *Rhodococcus equi* and commensals, which could potentially infect or colonize other animals [225]. We therefore suggest that more studies are warranted to simultaneously investigate the profiles and associations of resistome in the digestive tract of both food-producing and companion animals living in the same farm, in order to assess the potential risks of transmissions of ARGs among them and human beings.

## Conclusions

Based on metagenomic sequencing, it has been shown that there are abundant ARGs in the digestive tract of food-producing animals. The existence of these ARGs may not always be directly related to AMU, but is undoubtedly influenced by the use of injectable antimicrobials or their administration through feed or water. In most studies, feces were used to investigate the impact of antimicrobials on the diversity, profile and abundance of ARGs within the resistome. While fecal samples are easy to collect and are often used as a proxy of the microbial population within the digestive tract, composition of the microbiota differ across segments within the digestive tract [132, 163, 226, 227]. Additionally, compared to fecal microbiota, which originates from digesta, mucosa-associated microbiota can directly interact with the host where mucosa-associated microbiota are more likely to contact antimicrobials within the blood stream. It has also been shown that mucosa-associated and digesta-associated bacterial populations in the ileum of swine respond differently to antimicrobials [227]. It can therefore be speculated that, the response to the administration of antimicrobials may differ between mucosa- and digesta-associated microbiomes resulting in differences in associated resistome of these populations. In this regard, more investigations into the change in the resistome in mucosa-associated microbiota induced by antimicrobials in food-producing animals are warranted. In the future, it is necessary to further utilize emerging techniques for the analysis of resistome such as long-read sequencing, with special focuses on the expression of ARGs and the role of MGE in the dissemination of AMR in the gut of different food producing animals. Such information will be pivotal in defining the risk of spread of ARGs from food-producing animals to humans, and to develop effective strategies to reduce the threat of ARGs to human health and the environment.

## Data Availability

Not applicable

## Abbreviations

ACLAME: A CLAssification of Mobile genetic Elements
AMR: Antimicrobial resistance
AMU: Anti-microbial use
ARDB: Antibiotic Resistance Genes Database
ARGs: Antimicrobial resistance genes
ARGO: Antibiotic Resistance Genes Online
ARIBA: Antimicrobial Resistance Identification By Assembly
BLAST: Basic local alignment search tool
BWA: Burrows-Wheeler aligner
CARD: Comprehensive Antibiotic Resistance Database
GROOT: Graphing Resistance Out Of meTagenomes
HGT: Horizontal gene transfer
MAGs: Metagenomic-assembled genomes
MALDI-TOF: Matrix-assisted laser desorption ionization time-of-flight
MDR: Multidrug resistance
MGE: Mobille genomic element
MLS: Macrolide-lincosamide-streptogramin
NGS: Next-generation sequencing
PCR: Polymerase chain reaction
QC: Quality control
SiC-seq: Single-cell genome sequencing
SRST2: Short read sequencing typing
